# Supplementary material for: Resettlement willingness: From a village environmental perspective
Source: PLoS One. 2024 Aug 29;19(8):e0305476. doi: 10.1371/journal.pone.0305476 (PMC11361423; doi:10.1371/journal.pone.0305476)
Supplement: Attachment — Submitted filename: Point-to-point Response-2.docx [file pone.0305476.s003.docx]

1. We note that Figure 2, 3 and 6 in your submission contain [map/satellite] images which may be copyrighted. All PLOS content is published under the Creative Commons Attribution License (CC BY 4.0), which means that the manuscript, images, and Supporting Information files will be freely available online, and any third party is permitted to access, download, copy, distribute, and use these materials in any way, even commercially, with proper attribution. For these reasons, we cannot publish previously copyrighted maps or satellite images created using proprietary data, such as Google software (Google Maps, Street View, and Earth). For more information, see our copyright guidelines: http://journals.plos.org/plosone/s/licenses-and-copyright.

Response: The administrative boundary data and elevation data are from the Center for Resource and Environmental Sciences and Data of the Chinese Academy of Sciences (www.resdc.cn). According to the Resource and Environmental Sciences Data Registration and Publication System (https://www.resdc.cn/DOI/) data users can cite the results of this data as if it were a paper. The revised manuscript has cited the data as the 50th reference of the paper.

At the same time, we found that some of the papers published by PLOS one are also cited in this way. For example, "Spatial distribution and influencing factors of high-quality tourist attractions in Shandong Province, China" https ://doi.org/10.1371/journal.pone.0288472
